# Tbx3 represses PTEN and is over-expressed in head and neck squamous cell carcinoma

**DOI:** 10.1186/1471-2407-12-481

**Published:** 2012-10-19

**Authors:** Durmus Burgucu, Kenan Guney, Duygu Sahinturk, Irem Hicran Ozbudak, Deniz Ozel, Gulay Ozbilim, Ugur Yavuzer

**Affiliations:** 1 Department of Physiology, School of Medicine, Akdeniz University, Antalya, 07058, Turkey; 2 Department of Ear-Nose and Throat Head and Neck Surgery, School of Medicine, Akdeniz University, Antalya, 07058, Turkey; 3 Life Sciences Research and Application Centre, Akdeniz University, Antalya, 07058, Turkey; 4 Department of Pathology, School of Medicine, Akdeniz University, Antalya, 07058, Turkey; 5 Department of Biostatistics and Medical Informatics, School of Medicine, Akdeniz University, Antalya, 07058, Turkey

**Keywords:** Tbx3, PTEN, Cancer, Squamous cell carcinoma

## Abstract

**Background:**

Despite advances in diagnostic and treatment strategies, head and neck squamous cell cancer (HNSCC) constitutes one of the worst cancer types in terms of prognosis. PTEN is one of the tumour suppressors whose expression and/or activity have been found to be reduced in HNSCC, with rather low rates of mutations within the PTEN gene (6-8%). We reasoned that low expression levels of PTEN might be due to a transcriptional repression governed by an oncogene. Tbx2 and Tbx3, both of which are transcriptional repressors, have been found to be amplified or over-expressed in various cancer types. Thus, we hypothesize that Tbx3 may be over expressed in HNSCC and may repress PTEN, thus leading to cancer formation and/or progression.

**Methods:**

Using immunohistochemistry and quantitative PCR (qPCR), protein and mRNA levels of PTEN and Tbx3 were identified in samples excised from cancerous and adjacent normal tissues from 33 patients who were diagnosed with HNSCC. In addition, HeLa and HEK cell lines were transfected with a Tbx3 expressing plasmid and endogenous PTEN mRNA and protein levels were determined via qPCR and flow cytometry. Transcription assays were performed to demonstrate effects of Tbx3 on PTEN promoter activity. Mann–Whitney, Spearman’s Correlation and Wilcoxon signed-rank tests were used to analyze the data.

**Results:**

We demonstrate that in HNSCC samples, Tbx3 mRNA levels are increased with respect to their normal tissue counterparts (p<0.001), whereas PTEN mRNA levels are significantly reduced in cancer tissues. Moreover, Tbx3 protein is also increased in HNSCC tissue sections. Over-expression of Tbx3 in HeLa and HEK cell lines causes reduction in endogenous PTEN mRNA and protein levels. In addition, transcription activity assays reveal that Tbx3 is capable of repressing both the basal and induced promoter activity of PTEN.

**Conclusions:**

We show that Tbx3 is up-regulated in tissue samples of HNSCC patients and that Tbx3 represses PTEN transcription. Thus, our data not only reveals a new mechanism that may be important in cancer formation, but also suggests that Tbx3 can be used as a potential biomarker in cancer.

## Background

The T-box is a conserved DNA-binding and dimerization motif, which was first identified in the mouse protein Brachyury [1] and the genes encoding T-box containing proteins are collectively known as the T-box (TBX) family of genes. Tbx transcription factors family plays important roles in cell proliferation, fate and identity during development [2,3]. Based on their sequence similarities, five subfamilies have been identified in mouse and the Tbx2 subfamily is comprised of *Tbx2*, *Tbx3*, *Tbx4* and *Tbx5*[4]. Through their T-box domains, each T-box factor binds to the “T-half-site” found in the promoters of the target genes and regulates gene expression by either activating or repressing transcription. Amongst the Tbx family of proteins, Tbx3 and Tbx2 are known to function generally as transcriptional repressors, although Tbx3 has also been shown to have an activation domain [5-7]. In humans, *TBX3* mutations cause ulnar-mammary syndrome (UMS) which is characterized by mammary gland hypoplasia, abnormal limb development and various abnormalities of the heart and genitalia [8]. In addition to its key role in development, *TBX3* expression has also been found to be amplified or over-expressed in many different cancer types including breast, cervix, ovary, pancreas, liver cancers and melanomas [9-14]. Research towards identifying the molecular mechanisms of Tbx3 in cancer formation revealed that Tbx3 interacts with proteins of several oncogenic pathways. In liver tumorigenesis for example, Tbx3 lies downstream of the Wnt-β-catenin pathway and is a regulator of β-catenin [12]. Tbx3 also represses E-cadherin which has been implicated in metastasis of epithelial tumours [13]. In breast cancer, FGF signalling regulates Tbx3 [15] and Tbx3 cooperates with c-Myc and Ras associated transformation [16,17]. Another well-defined pathway that Tbx3 takes part is the p14/19^ARF^-Mdm2-p53 pathway. Tbx3 represses the expression of the human tumour suppressor gene p14^ARF^ and the murine homolog p19^ARF^[17-19]. In addition, Tbx3 directly represses the p21^*Cip1/WAF1*^ promoter [20]. The p14/19^ARF^-Mdm2-p53 pathway plays an important role in regulating cell senescence and protects the cells against oncogenic transformation. Repression of p14/19^ARF^ or p21^*Cip1/WAF1*^ by Tbx3 seems to block this protective pathway and bypass cellular senescence via p53 dependent or independent ways, thus leading to uncontrolled cell proliferation. In the light of the available evidence, it seems that Tbx3 uses multiple pathways and mechanisms in driving tumorigenesis.

Head and Neck cancers originate on the mucosal surfaces of oral cavity, pharynx and larynx. Because 90% of these malignancies exhibit squamous cell characteristics, the head and neck cancers are commonly called “Head and Neck Squamous Cell Carcinomas” (HNSCC). Worldwide, HNSCC is the sixth most common cancer type, with extremely poor clinical outcomes [21,22]. Research during the last decade revealed some of the molecular mechanisms underlying the pathogenesis of HNSCC. Inactivation of many tumour suppressor gene products such as p53, p16INK4a, E-cadherin and PTEN [23-27] or activation of proto-oncogenes such as Cyclin D, EGFR and p63 [28-30] have been found to be implicated in HNSCC occurrence.

The gene phosphatase and TENsin Homolog (PTEN) encodes a tumour suppressor which is mostly inactivated in many cancers. PTEN is the main negative regulator of the phosohatidylinositol-3-Kinase (PI3K) signalling pathway. PI3 kinases that are activated by either receptor tyrosine kinases (RTK) or G-protein coupled receptors (GPCR), catalyze conversion of phosohatidylinositol 4,5 phosphate (PIP2) to phosohatidylinositol 3,4,5 phosphate (PIP3), thereby activate AKT kinase and subsequent downstream components [31]. PTEN is a lipid and protein phosphatase and inhibits PI3K mediated signals involved in cellular growth, proliferation and survival by dephosphorylating PIP3 at the plasma membrane [32]. Although PTEN was found to be down-regulated in many different cancer types, this is not necessarily due to somatic mutations of the PTEN gene. Indeed, in a group of HNSCC tumour samples, it was demonstrated that down-regulation of PTEN was not due to its allelic loss or point mutations within the gene [26,27], indicating that the reduced expression of PTEN might be as a consequence of either transcriptional or post-transcriptional regulations.

The role of Tbx3 in regulation of pathways involved in cell proliferation, especially tumour suppressors such as E-cadherin and p53, which have also been found to be inactivated in HNSCC, prompted us to analyze the Tbx3 status in this particular type of cancer. To this end, in a period of 2 years, tissue samples were collected from patients undergoing operation with the diagnosis of HNSCC. During operations, samples were excised from both the seemingly cancerous areas and also from the normal-looking tissue surrounding the lesion site. Both kind of tissues (cancer and normal) were examined pathologically and then analyzed for expression of Tbx3 mRNA and protein levels. In addition, PTEN mRNA was also measured as it has been shown to be down-regulated in most of the samples of HNSCC. In this paper we report that in HNSCC samples both the mRNA and protein levels of Tbx3 are increased with respect to their normal tissue counterparts, whereas PTEN mRNA levels are decreased in cancer tissues as it has been reported before [33]. We also demonstrate that in two different cell lines, over expression of Tbx3 causes reduction in endogenous PTEN mRNA and protein levels. In addition, using transcription activity assays we show that Tbx3 is capable of repressing both the basal and induced promoter activity of PTEN.

## Methods

### Tissue samples

In a two-year period, surgical resection specimens from 33 patients who underwent partial or total laryngectomy for HNSCC in the Ear-Nose and Throat Head and Neck Surgery Department of Akdeniz University, School of Medicine, were collected. In compliance with the principles of the Declaration of Helsinki, the study was approved by the Ethical Committee of the University (No: 04.12.09/011398) and written informed consents were obtained from patients who accepted to participate in the study. During operation, samples were collected from both the cancerous area and the adjacent normal tissues. Before sending the tissues for pathological examination, about 10 mg from each of the tissue (cancer and normal) was spared and immediately placed in RNAlater TissueProtect Tubes (Qiagen GmbH, Cat No: 76163) to prevent degradation of RNA. The specimens were examined by the Pathology Department of the same University. Following routine paraffin embedding, sections in 5μm thicknesses were prepared and stained with haematoxylin/eosin (H&E) for histopathological evaluation. Pathological features of the patients were examined according to the American Joint Committee on Cancer (AJCC) [34]. Upon confirmation of tissues as cancerous and normal, the samples that were spared for RNA analyses were sent to the Physiology Department and stored at -80°C till usage.

### RNA analysis

Frozen tissues were disrupted and homogenized by MagNa Lyser Instrument (Roche, GmbH) and total RNA was isolated by using RNeasy Mini Kit (Qiagen GmbH, Cat No: 74124) according to the manufacturer’s instructions. RNA (1μg) was then reverse transcribed using the Transcriptor High Fidelity cDNA synthesis kit according to the manufacturer’s instructions (Roche GmbH, Cat No: 508195500). For real-time PCR, LightCycler 1.5 Instrument was used. The primer pairs for amplification of Tbx3 [GenBank:NM_005996.3], PTEN [GenBank: NM_000314.4**]** and human β-actin [GenBank:NM_001101.3] were designed using the Universal Probe Library (UPL) Assay Design Center ( http://www.roche-applied-science.com). The human β-actin gene was used as an internal standard to correct sample-to-sample variations within a PCR run. Hydrolysis (TaqMan) probes; Probe 47 (Roche GmbH, Cat no: 04688074001), Probe 48 (Roche GmbH, Cat no: 04688082001) and Probe 64 (Roche GmbH, Cat no: 04688635001) from the UPL were used for detection and quantification of Tbx3, PTEN and human β-actin mRNAs. The cDNA from each gene was amplified by PCR using the appropriate primer sets and probes with the TaqMan Master Mix (Roche GmbH, Cat No: 04735536001) according to the manufacturer’s instructions. The data was analyzed using the analysis module for absolute quantification of LightCycler Software 4.1.

### Construction of the PTEN promoter reporter plasmids

The PTEN promoter region [GenBank No: AF067844.1] between positions −1895 to + 400 (Extended promoter – EP-PTEN) was amplified from genomic DNA by PCR using the primers (F1) *5′-*agac**agatct**GTGGGGTGCGGGGTAGGAGT and (R1) 5′-agac**aagctt**GACGAAGAGGAGGCGAGA. For the amplification of the core promoter region (CP-PTEN) lying between −1477 and −710, the primer pair (F2) 5′-agac**agatct**GGCTTGCTCTTAGGGTAG and (R2).

5′-gcgt**aagctt**CGTGAACACATAGCCGT was used. The deletion mutant (Δmut CP-PTEN) between positions −1345 and −710 was amplified from genomic DNA via PCR using the F3 (5′- agac**agatct**CCAGTTCCCCAAGCGCCAG) and R2 primer pair. The sequences in lowercase are present to facilitate cloning by placing *Bgl II* and *Hind III* restriction enzyme recognition sequences (depicted in bold and underlined). The EP-PTEN (2295 bp), the CP-PTEN (767 bp) and the Δmut CP-PTEN (635 bp) PCR products were digested by *Bgl II/ HindIII* and cloned into a pGL3.1-Basic plasmid (Promega), which was linearized using the same restriction endonucleases. All constructs were verified by DNA sequencing. The Tbx3 and USF expression plasmids were kindly provided by Prof. Colin R. Goding (Ludwig Institute for Cancer Research, Oxford, UK).

### Cell culture and transcriptional activity assays

The HeLa and HEK cell lines were used for transcriptional activity assays as these cell lines are well known for being easy to grow and readily transfectable. In addition both cell lines express PTEN and do not contain genetic mutations of p53. The cell lines were maintained at 37^0^C with 5% CO_2_ in Dulbecco’s Modified Eagle Medium (DMEM) supplemented with 10% fetal calf serum (FCS) and 1% penicillin/streptomycin. Cells were plated on 96-well dishes and co-transfected with 0.5μg/well PTEN pGL3.1 reporter plasmids and Tbx3 and/or USF expression plasmids (0.2 to1.0 μg/well) using FuGene HD (Roche, Cat no: 04709691001) according to the manufacturer’s instructions. After 48 hours, cells were harvested and lysed by using the One-Glo Luciferase Assay System (Promega, Cat no:E6110). The lysates were then analyzed on a luminometer (Luminoskan Ascent, ThermoScientific). The experiments were repeated at least five times before establishing the final data. For each construct, values from 5 different experiments were obtained; average values were calculated and the data was presented as “% Promoter Activity” relative to the promoter activity of CP-PTEN (100%). Flow cytometry and western blots were performed in parallel to confirm the basal and over-expressed Tbx3 protein levels for each experiment and one representative western blot was shown in the relevant figures. To measure the endogenous PTEN mRNA levels, HeLa and HEK cell lines were transfected with a Tbx3 expressing plasmid in different concentrations and cells were harvested 48 hours following transfection. From transfected and untransfected cell lines, RNA was isolated and quantitative reverse transcription-polymerase chain reaction (qRT-PCR) was performed as described above. For endogenous PTEN protein determination, both of the cell lines were seeded onto 6-well dishes and transfected with Tbx3 expressing plasmid as described before. Forty-eight hours following transfection, cells were harvested and flow cytometry was performed as mentioned below.

### Immunohistochemistry and immunoblots

Parafin embedded, 5μm thick tissue sections were stained for Tbx3 protein using a monoclonal anti-Tbx3 antibody (ABCAM, Cat no: ab89220). The sections were analysed using standard avidin-biotin immunohistochemical methods according to the manufacturer’s instructions (Vector Laboratories, Burlingane, California). An anti IgG-antibody was used as control.

For immunoblots; transfected and untransfected cells were harvested and lysed in lysis buffer (150 mM NaCl, 50 mM Tris pH 8.0, 1.0 % NP40) and the protein content was determined by Bradford method. Equal amounts of protein was loaded onto 10% SDS-PAGE and separated by electrophoresis (80V, 2hr). This was followed by blotting onto nitrocellulose membranes. Immunoblotting was performed using anti-Tbx3 antibody at a dilution of 1:100. Immunoreactive bands were revealed by an ECL kit (Amersham) according to the manufacturer’s instructions.

### Flow cytometry

Transfected and untransfected cell lines were harvested and washed twice with phosphate buffered saline (PBS). BD Biosciences Cytofix/Cytoperm^TM^ Kit (Cat no: 554714) was used for fixation and permeabilization of cells according to manufacturer’s instructions. Cells were incubated for 30 min. at room temperature with anti-Tbx3 (ABCAM, Cat no: ab89220) and anti-PTEN (BD™ Phosflow 560002, labelled with PE-A) antibodies simultaneously, which were diluted in BD Biosciences Perm/Wash Buffer in 1:50 and 1:10 ratios, respectively. Following the washing steps, cells were incubated with a secondary antibody (DyLight 488, ABCAM, Cat no: ab96879) at a dilution of 1:2000 to enable detection of Tbx3. Analyses were performed on a BD FACS Canto II and protein expression of both Tbx3 and PTEN were measured in a total of 10,000 cells.

### Statistical analysis

The Statistical Package for the Social Sciences (SPSS) 18.0 software was used. Comparison of the mRNA levels between the tumour and normal tissues were performed using the Wilcoxon signed-rank test. Spearman’s Correlation test was used for evaluation of PTEN mRNA levels in response to over-expressed Tbx3. Mann–Whitney test was employed for interpretation of the in vitro transcriptional activity assays. Data are means of **±**SDs of five independent experiments and p**<**0.05 was considered statistically significant.

## Results

### Tbx3 mRNA and protein expression is increased in HNSCC

**Figure 1:**
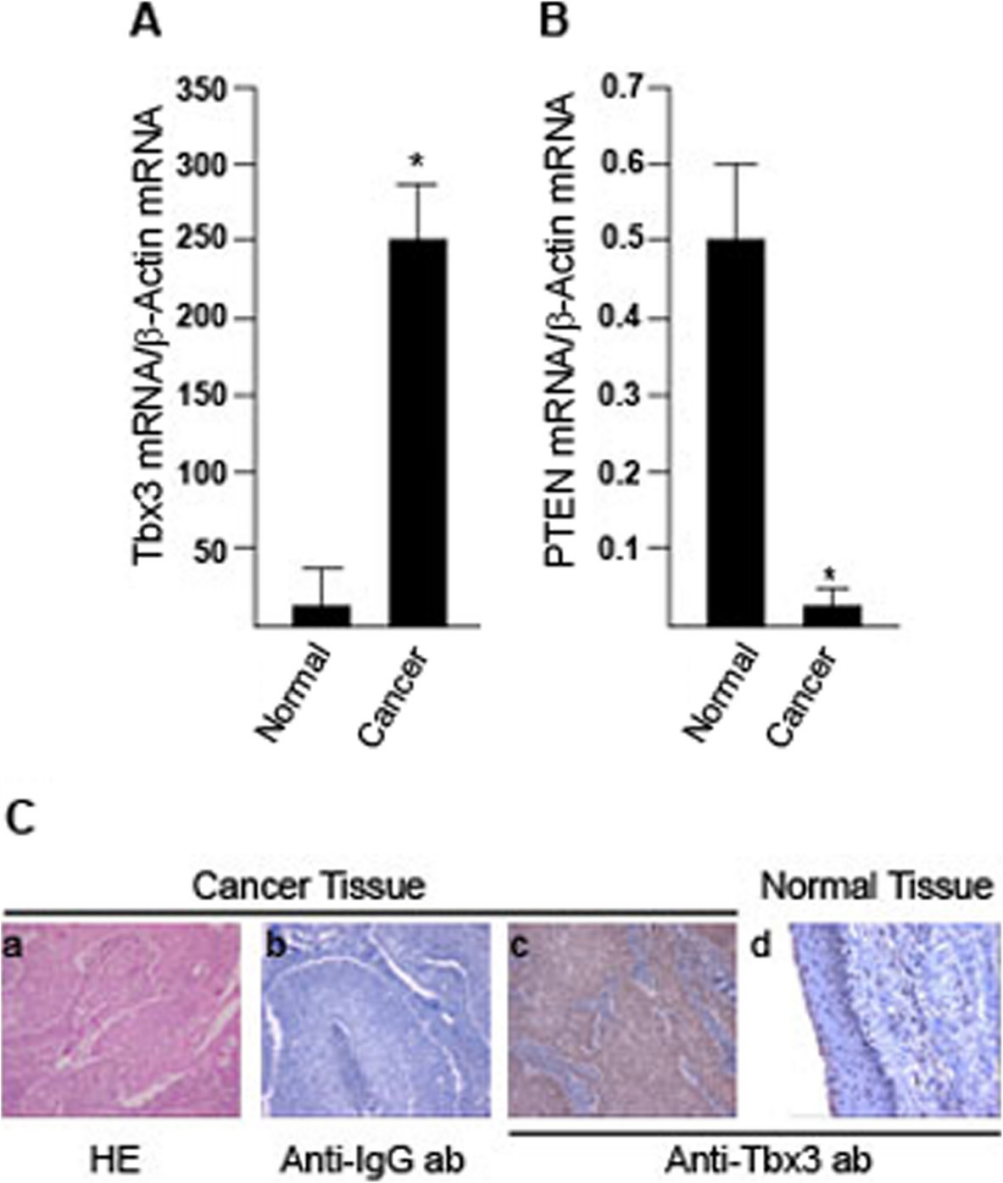
**Tbx3 mRNA and protein levels are increased in HNSCC tissues.** Tissues from cancerous and adjacent normal areas were excised from patients who were diagnosed with HNSCC. RNA was isolated and mRNA levels of Tbx3 (**A**) and PTEN (**B**) were quantified using TaqMan probes. Results were normalized to β-Actin mRNA levels. The sign “*”denotes for statistically significant increase and decrease with respect to the normal tissue mRNA levels (p<0.001). (**C**) Laryngeal squamous cell carcinoma tissue sections stained with haemotoxcylin/eosin (HE) (**a**) and anti-IgG antibody (**b**) as an isotypic control. Squamous cell carcinoma sections (**c**) exhibited stronger cytoplasmic and nuclear staining with anti-Tbx3 antibody with respect to the normal epithelium (**d**) (magnification 100x).

A total of 33 patients (32 males and 1 female), with a median age of 53 years, were analyzed. The majority of patients (85%) had HNSCC originated from larynx (n=28), 6% from tongue (n=2) and in 3 patients from oropharynx, submandibular gland or tonsils (3% each). The clinical stage was IV in 73% of patients, whereas 27% of patients were at stage III. In the sample group only one patient was at stage II. The mRNA expression of Tbx3 was found to be significantly increased in cancer samples (min-max: 22–7100, median: 242) with respect to the normal tissue (min-max: 12–1460, median: 5.38) obtained from the same individuals (p<0.001) (Figure 1A). Although generally PTEN mRNA expression was rather low in tissues, still there was a statistically significant reduction in expression in cancer tissues (min-max: 0–5.62, median: 0.0085) when compared to the normal tissue (min-max: 0–13.10, median: 0.5, p<0.001) (Figure 1B). Neither the clinical stage nor the origin of cancer exhibited a statistically significant difference in Tbx3 mRNA levels. In order to determine whether the increase in Tbx3 mRNA level correlates with protein level in HNSCC tissue samples, paraffin embedded cancer and normal tissue sections were stained with anti-Tbx3 antibody. As seen in Figure 1C, tumour sections (c) exhibited significantly stronger cytoplasmic and nuclear Tbx3 staining with respect to the normal tissues (d). We were not able to detect PTEN protein in HNSCC tissue samples, possibly due to low levels of PTEN mRNA in these tissues. As a consequence western blotting was not sensitive enough to detect such small amounts of protein. Nevertheless, these results demonstrated for the first time that both mRNA and protein levels of Tbx3 are increased in HNSCC tissue samples.

### Endogenous PTEN mRNA and protein levels are reduced in response to Tbx3 expression

**Figure 2:**
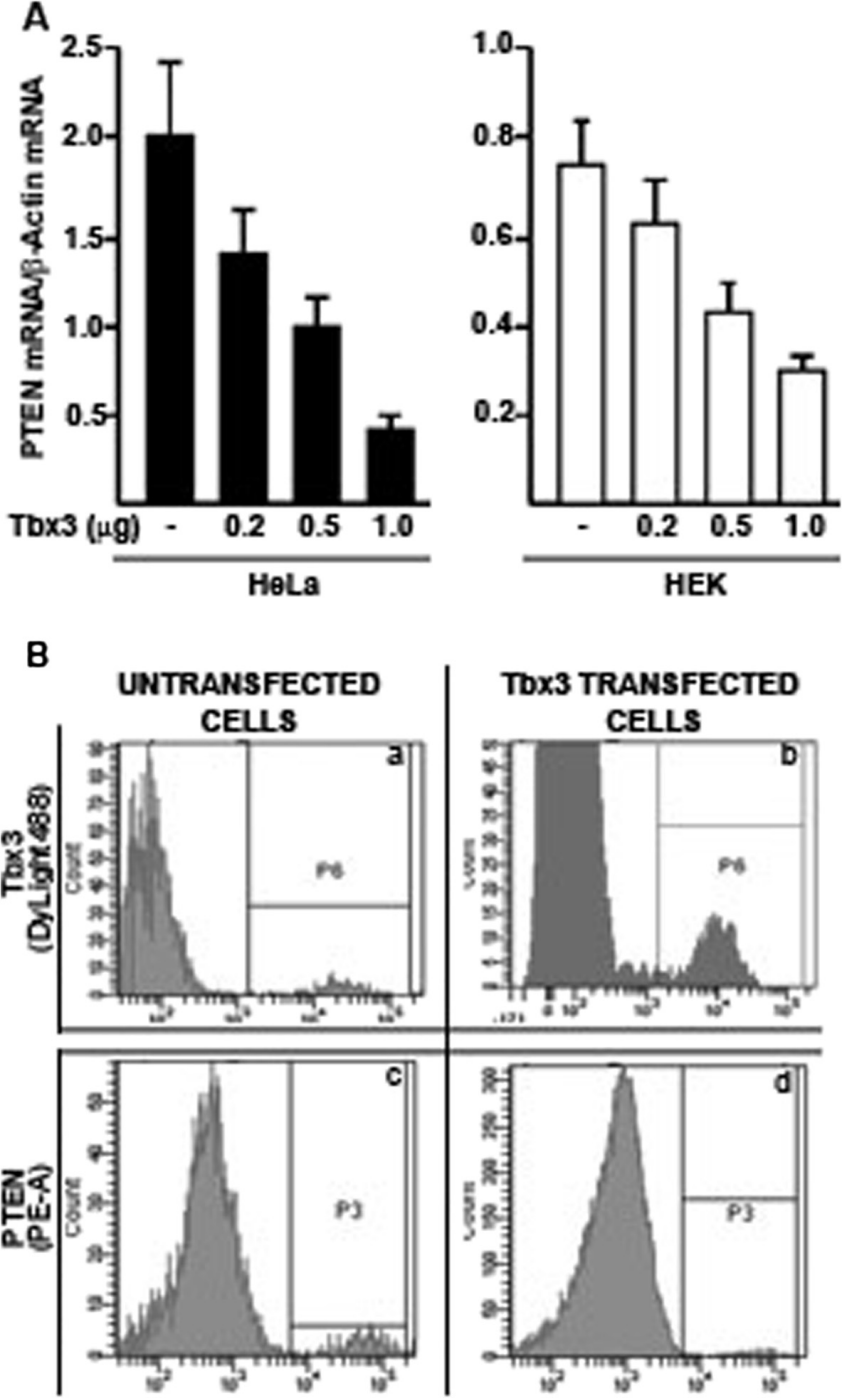
**Tbx3 represses endogenous PTEN mRNA levels.** (**A**) Different amounts of plasmid DNA (0.2, 0.5 and 1.0 μg) that expresses Tbx3 was transfected into HeLa and HEK cell lines. RNA from untransfected and transfected cell lines was isolated and PTEN mRNA levels were determined using TaqMan probes. Results were normalized to β-Actin mRNA levels. Spearman’s Correlation test revealed a significant decrease in PTEN mRNA levels with increasing amounts of Tbx3 expressing plasmid (r= −0.858 and p<0.05). (**B**) Cells transfected with 1μg of Tbx3 expressing plasmid and untransfected cells were analysed by flow cytometry using differentially labelled antibodies against Tbx3 (Labelled with DyLight 488) and PTEN (labelled with PE-A) for determination of Tbx3 and PTEN protein levels. Left panels (untransfected cells, **a** and **c**) display the endogenous Tbx3 and PTEN protein levels, which are shown in P6 and P3, respectively. Upon transfection, Tbx3 protein is increased (**b**) within the cells. In cells, where Tbx3 is over expressed, PTEN protein is decreased (**d**) with respect to that seen in untransfected cells (**c**).

The inverse correlation of Tbx3 and PTEN mRNA levels in HNSCC samples prompted us to analyze whether Tbx3 would cause a reduction in endogenous PTEN mRNA levels. To this end, HeLa and HEK cell lines were used as both of these cell lines are known to express PTEN and relatively low levels of Tbx3. The cell lines were transfected with a plasmid carrying a cDNA that expresses Tbx3. Three different plasmid DNA concentrations (as measured by a spectrophotometer) were used in transfections (0.2, 0.5 and 1 μg). Forty-eight hours following transfection, RNA was isolated from the transfected and untransfected cells and PTEN mRNA levels were quantified using TaqMan probes. As seen in Figure 2A, transfection of a Tbx3 expressing plasmid in increasing amounts caused a gradual decrease in PTEN mRNA levels in both of the cell lines. Spearman’s Correlation test revealed a significant negative correlation between the amounts of transfected Tbx3 expression plasmid and PTEN mRNA levels (r = −0,858 and p<0.05). In order to verify that transfection of a Tbx3 expressing plasmid causes over expression of Tbx3 protein in the cells, flow cytometry was employed. In addition, using a differentially labelled (PE-A) anti-PTEN antibody, PTEN protein levels were analysed in the same cell lines to determine whether over-expressed Tbx3 protein affects the PTEN protein level. In untransfected cells endogenous Tbx3 and PTEN levels were rather low (Figure 2B, panels a and c). Transfection of 1 μg of Tbx3 expressing plasmid caused an increase in Tbx3 protein levels (b) with respect to untransfected cells (a), demonstrating that Tbx3 protein was over-expressed in transfected cell lines (Figure 2B, panels a and b). Interestingly, cells transfected with a Tbx3 expressing plasmid displayed a reduction in PTEN protein levels with respect to the untransfected cells (Figure 2B, panels d and c, respectively). Thus, these results indicate that transfection of a Tbx3 expressing plasmid causes over expression of Tbx3 protein within the cells and the over-expressed Tbx3 protein results in reduction of both endogenous mRNA and protein levels of PTEN.

### Tbx3 Represses PTEN promoter activity

**Figure 3:**
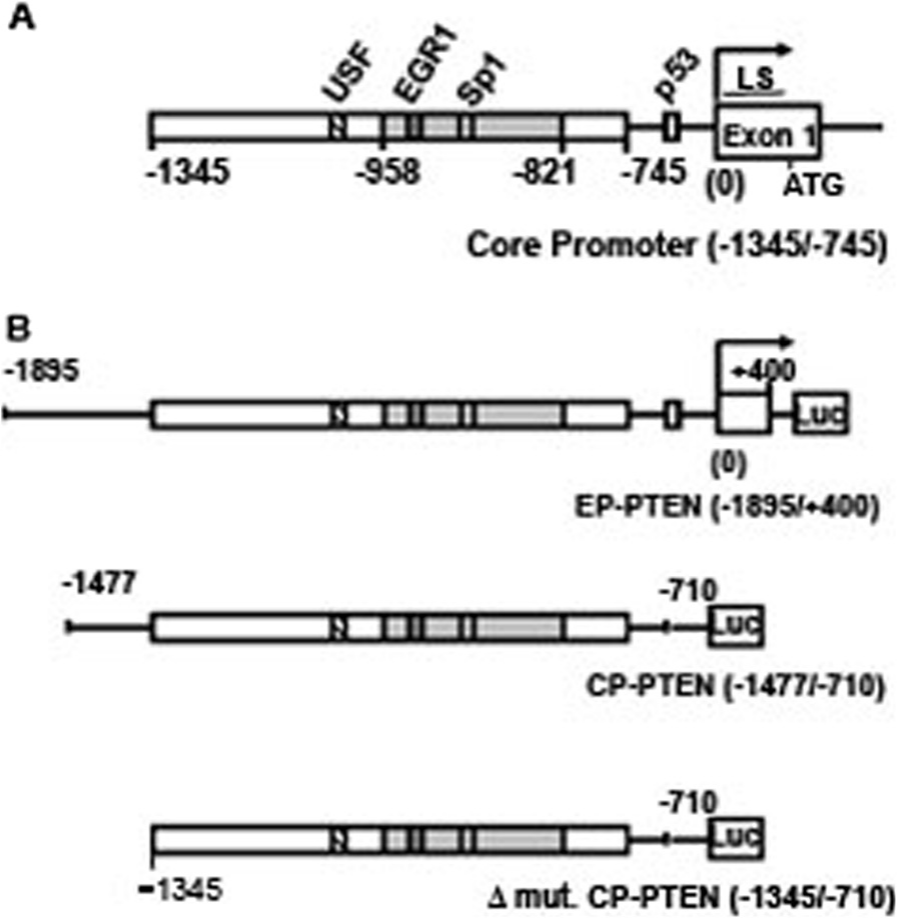
**Schematic diagram of PTEN promoter.** (**A**) Core promoter of PTEN localized between −1345 to −745 is shown. The beginning of exon 1 is designated as (0) and the start codon “ATG” within the first exon is indicated. A 1030 bp long leader sequence (LS) is found between the beginning of the first exon and the ATG site. The gray area between −958 and −821 is the minimal promoter of PTEN. Binding sites for the main transcription factors within the core promoter region are shown. (**B**) The EP-PTEN, CP-PTEN and Δ mut. CP-PTEN promoter constructs used in this study are shown. All promoter regions were cloned into the luciferase expressing plasmid pGL3 (Luc).

**Figure 4:**
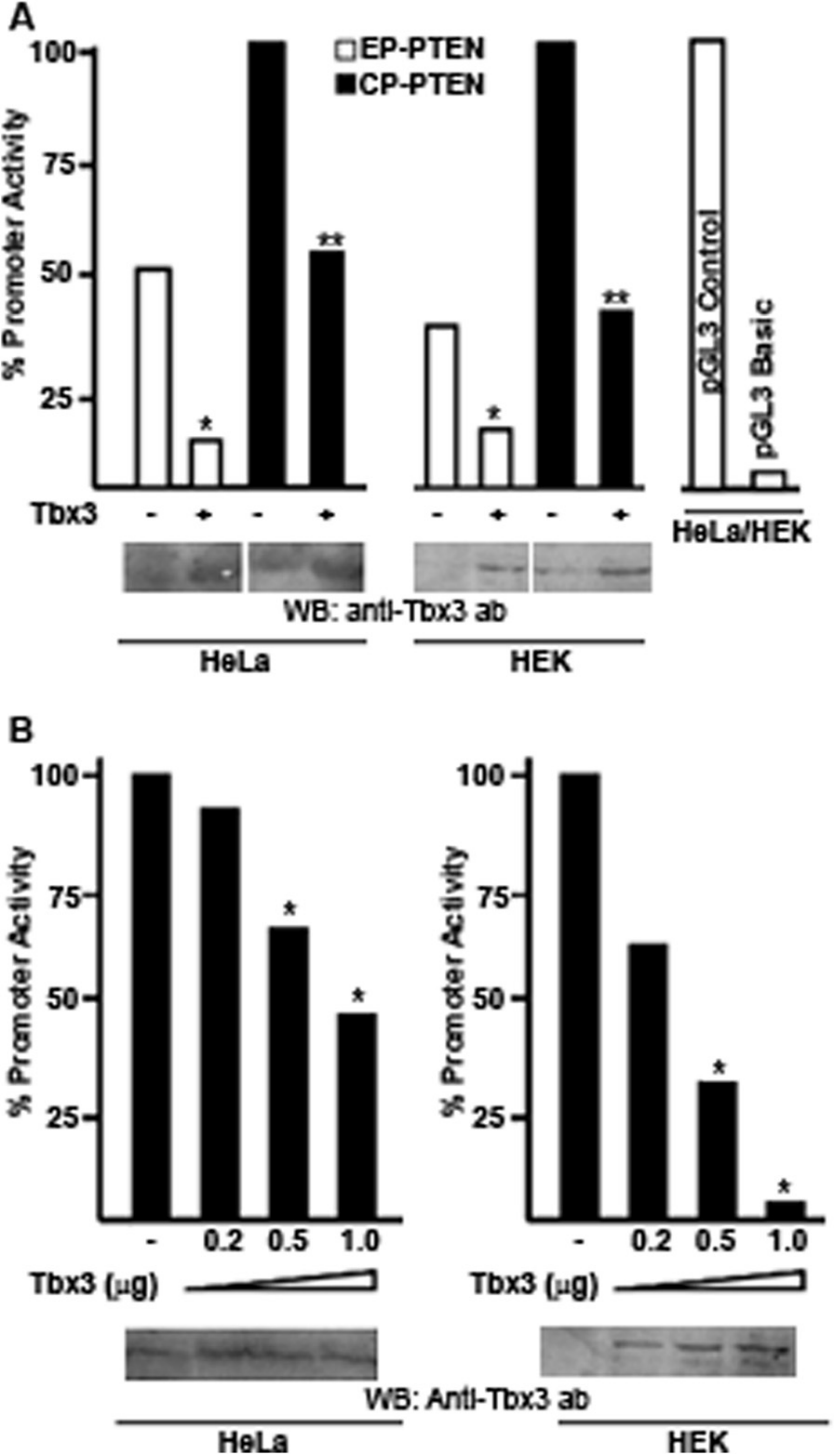
**Tbx3 represses PTEN promoter activity.** (**A**) EP-PTEN and CP-PTEN reporter plasmids were transfected into HeLa and HEK cell lines with (1μg) or without Tbx3 expressing plasmid. Using a luminometer, promoter activities of both plasmids were measured by means of luciferase activity. pGL3 control and pGL3 Basic plasmids were transfected into both of the cell lines as positive and negative controls, respectively. In both of the cell lines, expression of Tbx3 caused 3- to 4-fold reduction in the promoter activities of EP-PTEN and CP-PTEN reporter plasmids. Over expression of Tbx3 protein upon transfections was confirmed by western blots (WB) using an antibody against Tbx3 protein. (**B**) CP-PTEN reporter plasmid was transfected into both HeLa and HEK cell lines together with increasing amounts of a Tbx3 expressing plasmid. Promoter activity of CP-PTEN was then measured via luciferase assays using a luminometer. The sign “*”denotes for statistically significant decrease (p<0.05) in the PTEN promoter activity with respect to the basal activity of CP-PTEN as measured in the absence of Tbx3 expressing plasmid (100%). Expression of Tbx3 has been demonstrated by western blots (WB) using an anti-Tbx3 antibody.

The schematic diagram of the PTEN promoter region is given in Figure 3A. The analysis of the PTEN promoter [GenBank: AF067844.1] revealed that PTEN has a TATA-less and GC-rich promoter. The start codon (ATG) is preceded by a 1030 bp long leader sequence within the first exon. The core promoter of PTEN lies between positions −1344 to −745 with respect to the beginning of the first exon (0) [35]. This region contains binding sites for transcription factors USF, Sp1 and EGR1 and has been shown to govern maximum promoter activity [36-38]. The minimal promoter is also localized within this region between positions −958 to −821. In order to assess the role of Tbx3 in regulation of PTEN transcription, the PTEN promoter region between positions −1895 to +400 (EP-PTEN) and also a smaller region between −1477 and −710, encompassing the core promoter, were cloned into a luciferase reporter plasmid (CP-PTEN) (Figure 3B). Thus, the CP-PTEN encompassing the core promoter region extends about 100 bp upstream and 35 bp downstream from the core promoter of PTEN. Both of the PTEN promoter reporter plasmids were then transfected into the HeLa and HEK cell lines either on their own or together with a plasmid expressing Tbx3 (Figure 4A). In order to confirm over-expression of Tbx3, western blotting and flow cytometry were performed in parallel. As shown in Figure 4A, the promoter activity of CP-PTEN was 2-fold stronger than the EP-PTEN [35,39] and co-transfection of a Tbx3-expressing plasmid resulted in a 4-fold and a 2- fold reduction in the EP-PTEN and CP-PTEN promoter activities, respectively. Importantly, transfection of a Tbx3 expressing plasmid in increasing amounts, which causes over-expression of Tbx3 within the cells, resulted in progressive decrease in the PTEN promoter activity (Figure 4B). In both HeLa and HEK cell lines, a statistically significant decrease in the basal promoter activity of CP-PTEN was observed when 0.5 or 1 μg of Tbx3 expressing plasmid was transfected (p<0.05). However, especially in HeLa cells the CP-PTEN promoter did not exhibit a linear repression with increasing amounts of transfected DNA. In correlation with this, western blots did not demonstrate a linear accumulation of Tbx3 protein either. This could be due to inefficient translation process. In other words, probably every single transfected DNA molecule was not successfully translated into protein. Nevertheless, these results demonstrate that over-expression of Tbx3 within the cells results in repression of PTEN promoter activity.

**Figure 5:**
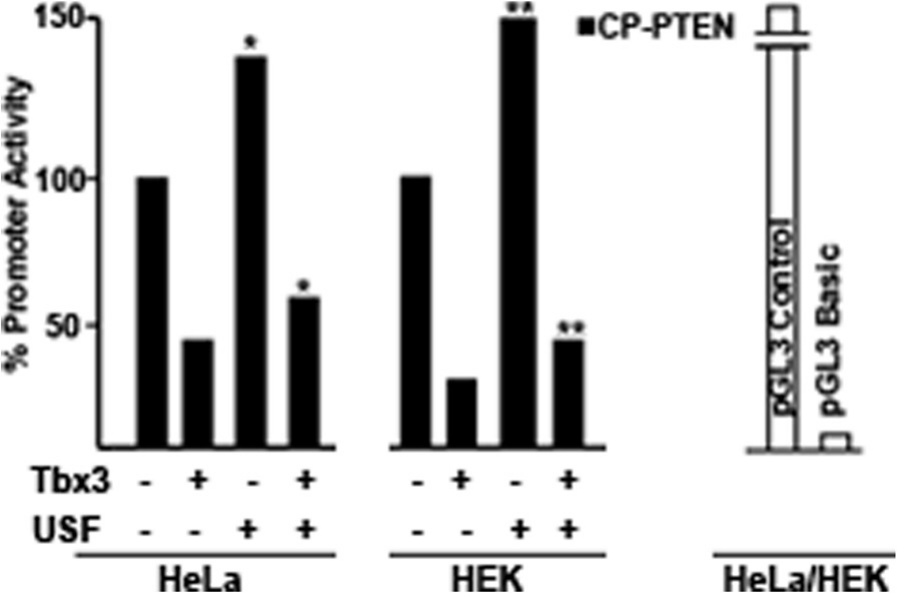
**Tbx3 represses induced PTEN promoter activity.** HeLa and HEK cell lines were transfected with the CP-PTEN reporter plasmid. Luciferase activity of CP-PTEN was measured in response to over expressed USF with or without Tbx3. USF is capable of inducing the transcriptional activity of CP-PTEN by 1.5-fold and Tbx3 is capable of repressing the induced transcription activity by 3-fold (p<0.05). pGL3 control and pGL3 Basic plasmids were transfected into both of the cell lines as positive and negative controls, respectively. The sign “*” denotes for statistically significant difference in PTEN promoter activity with respect to the basal activity (100%).

The positive acting transcription factor USF has been demonstrated to bind to a region within the core promoter of PTEN and induce the transcriptional activity of PTEN promoter [36]. In order to determine whether Tbx3 would have an effect on induced PTEN promoter activity, a USF expressing plasmid was transfected into HeLa and HEK cell lines. As expected, over-expression of USF caused 1.5-fold increase in the promoter activity of CP-PTEN (Figure 5). USF also increased the EP-PTEN promoter activity (data not shown). However, as shown in Figure 5, when Tbx3 was over-expressed alongside USF, it was still capable of repressing the induced transcriptional activity by 2.5- and 3-fold in HeLa and HEK cell lines, respectively (p<0.05). Thus, this data demonstrate that Tbx3 represses both the basal and induced PTEN promoter activity and that this repression is specific as similar patterns of repression was observed in two different cell lines.

### Tbx3 represses PTEN through a 132 bp DNA region within the PTEN promoter

**Figure 6:**
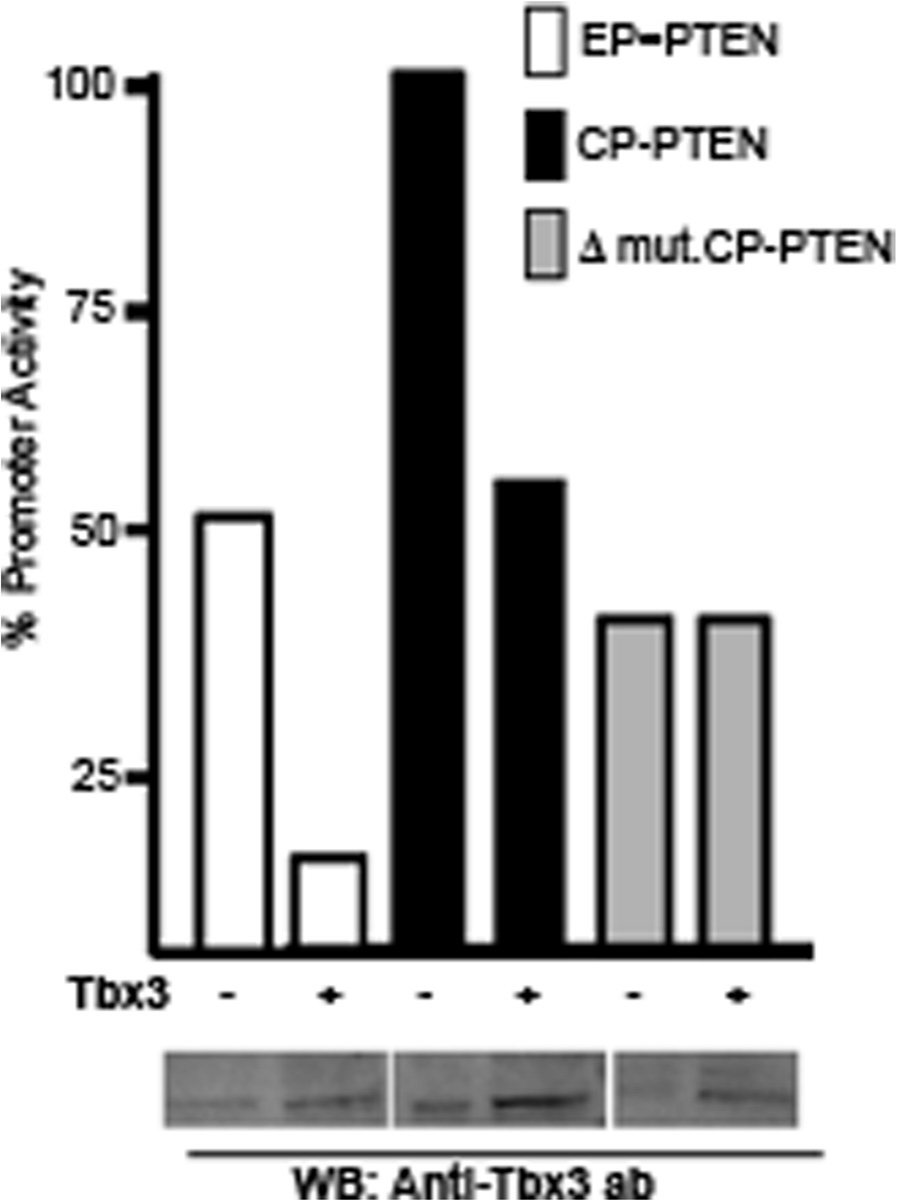
**A 132 bp DNA region is responsible for repression of PTEN promoter by Tbx3.** 132 bp from the 5^′^-end of CP-PTEN was deleted to generate the Δ mut. CP-PTEN. All three reporter constructs were transfected into HeLa cell line and promoter activities were determined in the absence or presence of 1μg Tbx3 expressing plasmid. As has been shown before, Tbx3 was capable of repressing promoter activities of both the EP-PTEN and CP-PTEN, whereas no repression by Tbx3 was detected in Δ mut. CP-PTEN. Western blot (WB) shown below was performed by an anti-Tbx3 antibody and displays the Tbx3 protein in untransfected and transfected cells.

Since both the EP-PTEN (−1895/+400) and CP-PTEN (−1477/-710) were repressed by Tbx3, we reasoned that the DNA region that is responsive to Tbx3 must be downstream of position −1477**.** In order to narrow down the Tbx3-responsive DNA region, we generated a third plasmid construct spanning between positions −1345 to −710 (Δ mut-CP-PTEN), in which 132 bp were deleted downstream of position −1477 (Figure 3A). This was then transfected into HeLa cells along with the two other PTEN reporter plasmids, EP-PTEN and CP-PTEN. As shown in Figure 6, basal activity of Δ mut-CP-PTEN was weaker compared to EP-PTEN or CP-PTEN, suggesting that the deleted 132 bp contains binding sites for positive acting factor/s. Interestingly however, although Tbx3 was capable of repressing EP-PTEN and CP-PTEN, the deletion mutant was not repressed by Tbx3. Analysis of this 132 bp DNA region did not reveal the presence of a canonical Tbx3 binding site. These results suggest that the PTEN promoter region between positions −1477 to −1345 contains binding sites for possible positive acting factors and Tbx3 represses PTEN promoter through this particular DNA region.

## Discussion

It is now believed that pathways that are critical for physiological development may also play a role in tumorigenesis. In support of this, many signalling pathways such as Wnt and Notch1 or transcription factors like Sox2, Tbx2 and Tbx3, all of which are important in embryologic development, were also shown to be involved in tumorigenesis [40]. Although Tbx3 exhibits an abnormal expression pattern in various cancers, molecular mechanisms underlying the role of Tbx3 in cancer are not yet entirely revealed [9-12,14]. Nonetheless, it is now clear that Tbx3 is one of the major proteins involved in pathways regulating cellular proliferation and senescence. In addition, accumulating evidence suggests that Tbx3 plays an essential part in metastasis [11,13,41]. This latter role seems to be very important as success in cancer treatment strategies largely depends on the capacity of primary tumour cells to metastasize.

In terms of prognosis, squamous cell cancer of head and neck constitutes one of the worst cancer types, despite advances in diagnostic and treatment strategies. Research towards identifying molecular mechanisms of HNSCC revealed that many oncogenes and tumour suppressors are involved in formation and progression of this particular type of cancer. Although these research allowed establishment of new treatment regimes targeting these signalling pathways, the prognosis is still rather poor due to the aggressive metastatic capacity of this cancer [42,43]. Metastasis of primary cancer cells to distant organs is believed to be governed by induction of a program called epithelial-to-mesenchymal transition (EMT) [44]. Recently, a microarray analysis performed on a panel of HNSCC cell lines demonstrated that in EMT-like HNSCC cell lines Tbx3 was one of the strongly up-regulated gene besides a set of 145 genes [45]. Although these results were obtained from cell lines, nevertheless they are in perfect agreement with our data presented in this paper which demonstrates that both the mRNA and protein levels of Tbx3 are increased in tissues obtained from patients with HNSCC. Humtsoe et al. [45] also showed that Tbx3 over-expression has resulted in EMT-like cell survival, while inhibition of Tbx3 by siRNA has suppressed cell invasion. These results indicate that Tbx3 expression in HNSCC cell lines induce metastasis and raise the possibility that Tbx3 may be an important diagnostic marker in HNSCC progression. However, in our study group neither the clinical stage nor the origin of cancer exhibited a correlation with the Tbx3 mRNA levels. This could simply be due to the relatively small sample size and the fact that in our samples the majority of HNSCC samples originated from larynx (85%) and the patients were at clinical stage IV (73%) or III (27%). It will be important to analyze Tbx3 mRNA and protein levels in larger groups and also in earlier stages of the disease.

Previously several mechanisms have been identified which Tbx3 uses during cancer formation and progression [12,16-20]. However, regulation by Tbx3 has not been demonstrated before for PI3K/PTEN/AKT signalling pathway, which plays very important roles in cancer formation, development and cancer cell metabolism. The protein kinase AKT is in the centre of PI3K/PTEN/AKT signalling pathway and induces cellular proliferation by regulating protein synthesis, cell metabolism and apoptosis. Activation of AKT is dependent on enhanced activity of PI3K or decreased activity of PTEN. Indeed, after p53, PTEN is the second most altered tumour suppressor in cancers. However, although a reduced expression of PTEN is observed in most of the solid tumours, genetic mutations of PTEN are rather rare in most cancer types, except in glioblastome multiforme and endometrial cancer [46]. In HNSCC, mutations or amplifications of the genes encoding PI3K or AKT2 have been identified [47-49], but although a reduced protein expression of PTEN has been reported [27,50-52], rather low rates of mutations (8%) within the PTEN gene were found [53]. Therefore, one of the reasons for the loss of PTEN activity or protein expression observed in HNSCC or other types of cancer could be through down-regulation of PTEN via transcriptional regulation. The core promoter of PTEN located at positions −1344 to −745 was found to be capable of governing the maximum promoter activity [35]. Several studies performed on PTEN promoter clearly revealed the presence of negative regulators located both upstream and downstream of the core promoter, however these regions were not analyzed further as they were not within the scope of the respective papers [35,36,39]. In this paper we demonstrated that Tbx3 is one of the repressors as it was capable of repressing the transcription activity of PTEN promoter in both HeLa and HEK cell lines. In addition, when the PTEN core promoter activity was induced by over-expressing USF, Tbx3 was again able to overcome the transcriptional activity and repress the induced PTEN promoter activity. The repression of the induced PTEN promoter activity by Tbx3 could be due to the binding of Tbx3 and USF to the PTEN promoter simultaneously. However, it is also entirely possible that over-expressed Tbx3 could have down-regulated USF expression. Even so, these results and the fact that increasing amounts of Tbx3 causes a gradual decrease in the promoter activity of PTEN imply that Tbx3 is capable of repressing PTEN and that this repression is specific.

How and where within the PTEN promoter Tbx3 binds to, is not yet clear. Since the deletion mutant (Δ mut CP-PTEN) was not repressed by Tbx3, it can be argued that the Tbx3 responsive DNA region within the PTEN promoter is localized between positions −1477 and −1345. Unfortunately, search for a Tbx3-protein binding site within this 132 bp region did not reveal a consensus binding site. However, it is known that although many of the Tbx-family of proteins bind to brachyury site [54] either using the whole palindrome or to half-sites only, there can be some variations within the consensus sequence [55]. It is also noteworthy to mention that DNA-binding may not necessarily be an intrinsic determinant for specificity for Tbx-family of proteins. Indeed, accumulating evidence indicates that for Tbx family of proteins, DNA target specificity is dictated by interactions with other transcription factors and specific chromatin determinants [56-58]. Thus, identification of Tbx3 binding site within the PTEN promoter or any possible interactions of Tbx3 with the chromatin structure around the PTEN promoter require further research.

We demonstrated repression of PTEN transcription by Tbx3, both by using *in vitro* transcriptional activity assays and also by quantitative PCR, where we measured endogenous PTEN mRNA levels in response to over expressed Tbx3. As has been demonstrated by flow cytometry analyses, over expression of Tbx3 also caused a reduction in endogenous PTEN protein levels. Although reductions in endogenous PTEN mRNA and protein levels in response to over-expressed Tbx3 strongly suggest that this repression may occur *in vivo*, we were not able to demonstrate direct binding of Tbx3 to PTEN promoter *in vivo*. However, considering that Tbx3 has been implicated in metastasis of cervix, breast, head and neck squamous cell carcinomas, as well as melanomas [11,13,41,45], it is tempting to speculate that this repression may take place *in vivo* as well, especially during metastasis*.* Metastasis is the main cause of death in the majority of human cancers and in order to survive, cancer cells must overcome the challenges that metastatic processes present, such as apoptosis either due to cellular detachment or cell shape change [59]. Although resistance to apoptosis enables tumour cells to survive, it leads to a period of tumour dormancy, as growth in the metastatic sites is installed temporarily [60]. Anoikis is the term used to describe apoptosis due to cellular detachment. Role of PTEN in anoikis has been reported before [61] but direct effect of PTEN on anoikis and tumour dormancy has been demonstrated in mammary epithelial cell lines by disruption of PTEN expression using homologous recombination, which resulted in growth factor independent proliferation and resistance to anoikis [62]. Interestingly, in HNSCC cell lines showing EMT-like features in which Tbx3 was found to be over-expressed, it was also demonstrated that these cell-lines exhibited resistance to anoikis [45]. Therefore, it is possible that repression of PTEN by Tbx3 may account for resistance to anoikis observed in cancer cells.

## Conclusions

We have shown that Tbx3 is up-regulated in tissue samples obtained from patients with HNSCC. In addition, we demonstrated that Tbx3 is capable of repressing PTEN transcription. This repression may have implications in progression and metastasis of cancer cells. In this scenario, over-expression of Tbx3 may render the cancer cells to gain the metastatic capacity and by inhibiting PTEN, may enable cells to resist apoptosis, therefore giving them the chance to survive and migrate to distant sites.

## Abbreviations

DNA: Deoxyribonucleic acid; mRNA: Messenger ribonucleic acid; PCR: Polymerase chain reaction; bp: Base-pair.

## Competing interests

The authors declare that they have no competing interests.

## Authors’ contributions

DB carried out all molecular biology experiments and participated in statistical analysis. KG participated in the design of the study, carried out diagnosis and operations of the patients and sample collection during operations. DS carried out RNA extractions. IHO and GO carried out the pathological examinations and immunohistochemistry. DO performed the statistical analysis. UY designed and coordinated the study, analyzed the data and wrote the manuscript. All authors read and approved the final manuscript.

## Pre-publication history

The pre-publication history for this paper can be accessed here:

http://www.biomedcentral.com/1471-2407/12/481/prepub
